# Effective capture of As(V) from water by a facile one step hydrothermal synthesized of 2-D bismuthene quantum dots nanosorbent

**DOI:** 10.1186/s13065-024-01308-x

**Published:** 2024-10-17

**Authors:** Saad S. M. Hassan, Mohamed E. Mahmoud, Rana M. Tharwat, Amir M. Abdelfattah

**Affiliations:** 1 Faculty of Science, Chemistry Department, Ain Shams University, P.O. Box 80205, Cairo, Egypt; 2 Faculty of Sciences, Chemistry Department, Alexandria University, Moharem Bey, Alexandria, Egypt

**Keywords:** Bismuthene quantum dots, As(v), Adsorptive capture, Application

## Abstract

Arsenic species have been known for their toxic impact on human. Therefore, removal of such pollutant requires efficient and effective removal methodology from polluted water. In this study, bismuthene quantum dots (Bi-ene-QDs) were fabricated by a green and facile one pot-hydrothermal conversion reaction of Bi(NO_3_)_3_·5H_2_O. Bi-ene-QDs exhibited semi-spherical crystalline providing 6.0 nm 157.78 m^2^/g. Consequently, As(V) capturing by Bi-ene-QDs revealed optimum practical conditions at pH 3, interaction duration time 40 min and 10 mg Bi-ene-QDs dosage. The interaction of As(V) ions with Bi-ene-QDs were confirmed by the appearance of As-O stretching vibration. Moreover, Bi-ene-QDs achieved excellent adsorptive capture percentages of Arsenic ions from sea, tap and wastewater providing 94.61, 95.21 and 94.38% from contaminated samples with 5 mg L^−1^ Arsenic ions. Therefore, Bi-ene-QDs can be categorized as an unprecedented and efficient nanosorbent for the successful removal of Arsenic ions pollution from various wastewater matrices with > 90.0% efficiency.

**Supplementary Information:**

The online version contains supplementary material available at 10.1186/s13065-024-01308-x.

## Introduction

The evolution of nanotechnology has grabbed great scientific and technical attentions due to multiple implementations in various scopes as medicine, chemistry, physics and engineering [1–3]. Quantum dots are defined as synthetic materials from very tiny size less than 10 nm with extraordinary optical, electronic specifications and others which are considered a major theme in nanotechnology [4]. QDs are categorized into three classes as core-QDs, core–shell QDs and alloyed QDs as the QDs characteristics are not only detected with the particle size but also by constituents and formula [5]. Furthermore, core-QDs are defined as synthesized QDs from single uniformly distributed composite in the inner composition as selenides, sulfides and tellurides of transition metals besides carbon, graphene QDs silicon, germanium QDs and etc. [1]. However, core–shell QDs are known as QDs with modified optical specifications due to the formation of shells on the QDs core such as CdSe QDs in the center and ZnS QDs in the shell. Alloyed QDs are defined as multi composites alloyed QDs and generally utilized to fabricate QDs with elevated luminescence and improved optical and electronic characteristics without changing the molecular size like cadmium selenium sulfide with uniform and gradient inner formula [6]. Further, numerous researches investigated the QDs specifications and applications in several scopes as medicine for diagnosis and the detection of diseases by biosensors [7], besides electrochemical sensing which is dependent on the semiconductor QDs and their applications in ecological and food analysis [8]. Moreover, the specific optical characteristics of QDs as powerful photoluminescence permit them to be utilized in fluorescence picturing, light releasing diodes with various releasing spectra and solar cells [9, 10]. Thereafter, quantum dots (QDs) and their unique composites were recently employed in water remediation, besides other emerging applications [11]. QDs and the modulated compounds are also characterized with multiple emerging specifications, if compared to ordinary adsorbents as they have elevated sensitivity, and enhanced selectivity for sensing implementations [12, 13].

Hence, various focused studies were reported on the fabrication of quantum dots from diverse sources and their modification with multi-component systems to enhance the stability properties, elimination efficiency and minimization of toxicity in water remediation [14–19].

Bismuth (Bi) is one of the most explored elements of group 5A due to unparalleled characteristics as elevated electron movement (electron transmitting specifications), semi-metal binding, spin orbit reaction, elevated adaptability, low toxicity and high settlement [20]. In addition, compounds including bismuth attained high elimination efficiency for inorganic anionic pollutants by ligand interchange and electrostatic conjunction [21, 22]. The extremely small zero-dimensional bismuthene quantum dots (Bi-ene-QDs) material is known as a promising subordinate of 2D bismuthene with multiple characteristics to facilitate incorporation into fiber apparatus which showed great thermal and chemical settlement as well as in the field of ultrafast photonic fabrication [23–25]. However, the applications of Bi-ene-QDs derivatives in water treatment need to be widely investigated [22].

Arsenic is one of the most common poisons in the history due its high toxicity [26]. Therefore, ecosystem arsenic pollution is caused by naturalistic or anthropogenic way [27]. Naturalistic way involves volcanic eruptions, fires, and hydrothermal metallic sedimentation [28], while, anthropogenic way involve mining, besides various kinds of industrial operations like combustion of fossil fuels, agronomical production, iron and steel manufacture as well as tanning processes [29]. Exposition to arsenic can cause cancer to skin, kidney, bladder, and lung, besides stomach and esophageal pain, hyperkeratosis the increasing in the thickness of the skin, heart problems, liver disorder and cardiovascular diseases [30–33]. Unfortunately, around 50 million persons in Asia consumed arsenic with concentration 50 μg L^−1^ or more and about 500,000 of them died because of arsenic poisoning and consequently, (WHO) and (USEPA) recommended that the total arsenic concentration in portable water should not exceed 0.01 mg/L [34, 35].

Now a day, the arsenate decontamination as well as other pollutants from water has become a very important mission and therefore, several methods have been studied [36–42]. Nevertheless, these water purification technologies are suffering from multiple potential disadvantages as the necessity of effective and accurate pH adjusting, occurrence of interfering ions which decrease the arsenic elimination capacity, repeated recycling processes, besides expensiveness and the toxic solid reaction residuals [40]. Adsorption is considered the most convenient arsenic elimination method which is facile process, cheap, with high selectivity besides the broad availability of several adsorbents [43]. Several natural and artificial adsorbents have been testified for detecting the standard arsenic elimination conditions as carbon containing materials, clays and zeolites [44]. Nevertheless, these adsorbents have many defects as slow kinetic of elimination reactions, low elimination efficiency for high concentration and un-preferred cost which limit their wide application [45]. As previously reported, bismuth including biochar achieved was reported with good elimination capacity of arsenic [20], while bismuth oxide was identified to eliminate arsenite as well as arsenate [46].

Based on the above-mentioned facts, new, simple and effective adsorbent materials are recently aimed to design, assemble and investigate in removal of arsenic ions from aquatic systems. Moreover, sorbents including bismuthene quantum dots (Bi-ene-QDs) or their composites have not been widely investigated in water purification [47]. Thus, Bi-ene-QDs material was fabricated in this work by using a simple hydrothermal technique and aimed to apply as a promising nanosorbent for removal of As(V) ions based on the incorporated good as strong capture specifications with inorganic anions as arsenate. Consequently, the fabricated Bi-ene-QDs material was aimed to characterize by different technique to favor efficient arsenate removal with high percentages. The optimum conditions for uptake of As(V) were also aimed to figure out and optimize in this study.

## Experimental

### Instrumentation

All used equipment are listed in Table 1

**Table 1:** Instruments and specifications

Characterization technique	Instrument
The Fourier transform infrared (FTIR) spectra	BRUKER VERTEX 70 FT-IR spectrophotometer at 400–4500 cm^−1^
The X-ray diffraction(XRD) patterns	XRD Shimadzu lab X6100, Japan
X-ray photoelectron spectroscopy (XPS)	Thermo Fisher Scientific (UK) supported instrument with X-ray source gun A = X-Ray 002 400um—FG ON (400 µm)
Scanning electron microscopy (SEM) pictures besides the elemental composition of adsorbent	JSM-IT200 instrument with Energy Dispersive X-ray spectroscopy (EDX) were utilized after doping the sample by ion sputtering coating instrument (JEOL-JFC-1100E)
The pictures of transmission electron microscopy (TEM)	JEOL-JSM-1400Plus, Japan
Thermal gravimetric analysis (TGA)	TGA-50-Schimadzu, Japan
The Brunauer–Emmett–Teller (BET)	BELSORP-mini II, BEL Japan
The pH measurements	Were adjusted by Adwa PH-meter
UV–Visible spectrophotometer	UV–Visible 2700 adsorption spectrophotometer

### Fabrication of bismuthene quantum dots (bi-ene-qds)

Bi-ene-QDs were fabricated by facile one step hydrothermal process. Primarily, 30 mg of Bi(NO_3_)_3_·5H_2_O was added to 40 mL double distilled water (DDW) and stirred for half hour, then the mixture was transferred to a Teflon padded stainless steel autoclave and heated for 3 h at 180 °C to confirming the successful growth of bismuthene quantum dots. Subsequently, the autoclave was left to cool till reaching the room temperature and the resulted Bi-ene-QDs material was washed and subjected to centrifugation several times by DDW and then desiccated at 60 °C [48].

### As(V) removal studies

As(V) (1000 mg L^−1^) stock solution was initially prepared and 5, 10 and 25 mg L^−1^ solution were obtained by successive dilutions. The absorbance of As(V) was measured by a spectrophotometer at wavelength λ_max_ = 840 nm. Accordingly, 2 mL As(V) solution was mixed with 0.4 mL (6.5 g L^−1^) (NH_4_)_2_MoO_4_ and 0.2 mL of (100 g L^−1^) C_6_H_8_O_6_ then 10 mL DW was added and absorbance was determine after 90 min [49]. The As(V) removal from water onto Bi-ene-QDs were examined and optimized by several parameters by monitoring the related factors to Bi-ene-QDs) dosage, As(V) concentration, interaction duration, pH, ionic strength in addition to reaction temperature according to the following procedures.

#### The ph influence on As(V) removal by Bi-ene-QD

This factor was investigated by controlling the pH of 20 mL As(V) solutions (5, 10 and 25 mg. L ^−1^) concentrations by 0.1 M NaOH and 0.1M HCl at the pH range from pH 1.0 to 11.0. These were mixed with 10 mg of Bi-ene-QDs and mechanically vibrated for 40 min by automatic shaker. Eventually, Bi-ene-QDs were removed from solution by filter paper and the absorbance of residual As(V) was examined by a spectrophotometer. Moreover, point of zero charge (PZC) of Bi-ene-QDs was done by adding 0.1 g of Bi-ene-QDs to 40 mL of 0.01 potassium chloride, while the pH was adjusted at the extent 2–11 and the solutions were mechanically vibrated for four hours at room temperature. Thereafter, the eventual solutions pH was measured after 12 h and (PZC) was measured via plotting of ΔpH against initial pH.

#### The Bi-ene-QD dosage influence on As(V) removal

This factor was investigated by adjusting 20 mL of As(V) solutions (5, 10 and 25 mg L^−1^) to pH 3.0. Various dosages of Bi-ene- QDs in the rang 2–50 mg were then added and theses solutions were vibrated for 40 min. Eventually, posterior filtration the absorbance was measured as previously mentioned.

#### The ionic strength influence on As(V) removal

This factor was examined by adding 10 mg of Bi-ene-QDs to several weights of NaCl in the extent 10 -100 mg. it was then mixed with 20 mL of As(V) solutions (5, 10 and 25 mg L^−1^). After controlling the pH to 3.0, the solutions were mechanically vibrated for 40 min. Thereafter, the absorbance was detected after Bi-ene-QDs separation from solutions as previously mentioned.

#### The interaction duration influence on As(V) removal

This factor was examined by adding 20 mL of As(V) solutions (5, 10 and 25 mg L^−1^ to 10 mg of Bi-ene-QDs after controlling the solution pH to 3.0. These solutions were mechanically vibrated for the selected time period from 2 -60 min. Finally, the absorbance was detected as previously reported after Bi-ene-QDs separation from the solutions.

#### The As(V) concentration influence

This factor was examined by adding 10 mg Bi-ene-QDs to 20 mL As(V) solutions (5–50 mg L^−1^). After adjusting at pH3.0, the solutions were mechanically vibrated for 40 min. Then, the absorbance was detected after Bi-ene-QDs separation from solutions as previously mentioned.

#### The temperature influence on the As(V) removal

This factor was examined by adding 10 mg Bi-ene-QDs to 20 mL of arsenate solution (5, 10 and 25 mg L^−1^) at pH 3. These solutions were vibrated for 40 min after the temperature was thermostated to the extent 25 to 60 °C. Thereafter, the absorbance was detected after separation of Bi-ene-QDs from solutions as previously reported.

### Recycling of Bi-ene-QD

250 mg of Bi-ene-QDs was added to 40 mL of 100 mg L ^−1^ arsenate solution and mechanically vibrated for 40 min, then filtered and treated with 100 of 0.1M of hydrochloric acid in addition to DW to attaining neutral pH and eventually dried at 60  C. Subsequently,10 mg of Bi-ene-QDs was added to 20 mL arsenate solution (5, 10 and 25 mg L^−1^) and vibrated for 40 min. then the remaining arsenate concentration was determined as previously reported, and this work was repeated for two more cycles.

### Bi-ene-QD application to adsorptive capture of as(V) from actual water specimens

Bi-ene-QDs were applied to adsorptive capture of arsenate from real water matrices of tap water, wastewater and sea water to testify its efficiency. 10 mg mass of Bi-ene- QDs was reacted with 20 mL of 5, 10 and 25 mg L^−1^ As(V) that prepared by these specimens. These were then mechanically vibrated for 40 min. then the remaining arsenate concentration was determined as previously reported.

## Results and discussion

### Characterization

**Fig. 1:**
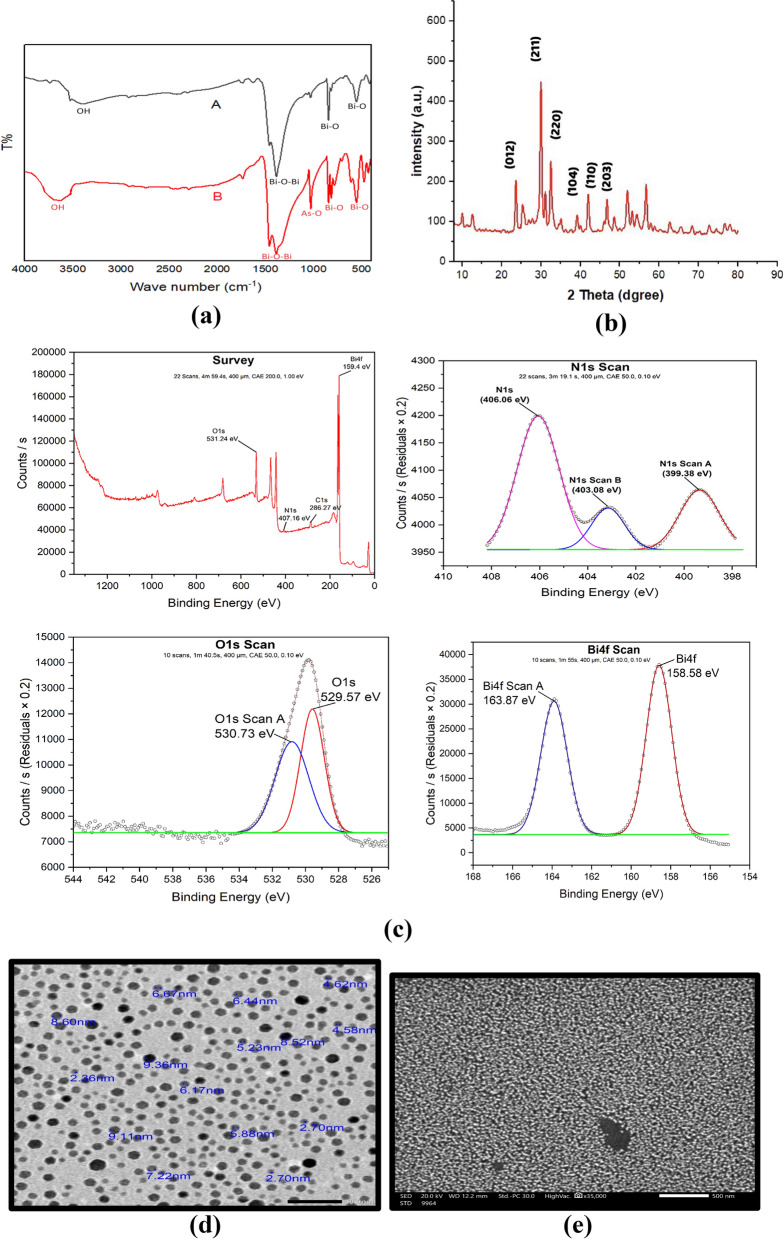

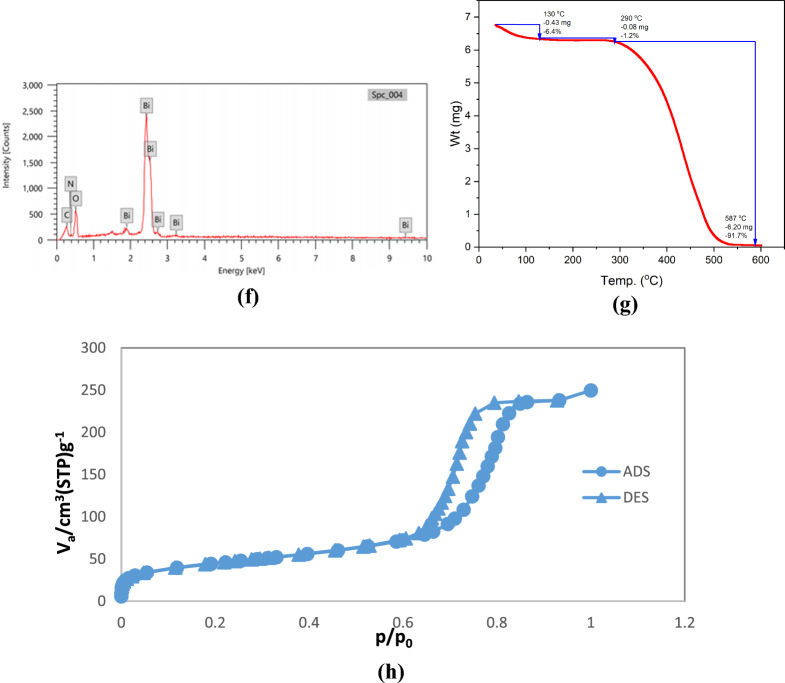
**a** FT-IR spectra of (A) Bi-ene-QDs and (B) As(V)@Bi-ene-QDs. **b** XRD patterns of Bi-ene-QDs. **c** XPS of Bi-ene-QDs. **d** TEM image of Bi-ene-QDs. **e** SEM image of Bi-ene-QDs. **f** EDX of Bi-ene-QDs. **g **TGA of Bi-ene-QDs**. h** N_2_ adsorption–desorption isotherm of Bi-ene-QDs

According to the FT-IR spectrum of Bi-ene-QDs illustrated in Fig. 1a a vibrational peak at 3394 cm^−1^ for the hydroxyl group present on the Bi-ene-QDs exterior. While the vibrational peak at 1385 cm^−1^ is confirming the presence of bismuth in Bi-ene-QDs and two other peaks at 844 cm^−1^ and 553 cm^−1^ are ascribed to the Bi-O bond vibration [50]. Furthermore, the FT-IR corresponding to As(V)@Bi-ene-QDs referred to a band at 1028 cm^−1^ corresponds to As-O stretching vibration based on the possible capture of As(V) with Bi-ene-QDs [51].

As clarified in Fig. 1b, the various bands in XRD pattern distinguish several bands at 25.29°, 39.13°, 41.97° and 46.69° which are conformable to planes (012), (104), (110) and (203) of the Bi-ene-QDs are perfectly matched with the rhombohedral Bi crystal formula (PDF#44e1246) [22]. While the two bands at 30.5° and 32.48° are correlated to the planes (211) and (220) to indicate the occurrence of Bi_2_O_3_ formula (PDF #78-1793) that was formed at the surface of Bi-ene-QDs to provide [52]. Therefore, it is evident from the XRD data that Bi-ene-QDs were successfully formed with crystalline formula.

The XPS survey of Bi-ene-QDs is provided in Fig. 1c to confirm the presence of Bi, O and N elements with atomic percentages 63.55%, 29.93% and 6.53%, correspondingly. The 158.58 eV and 163.87 eV peaks are indicating the chemical forms of Bi-4f_7/2_ and Bi-4f_5/2_, while the 159.4 eV band indicating the presence of Bi_2_O_3_ due to partially oxidized Bi-ene-QDs [53, 54]. The O 1s bands at 529.3 eV, 530.5 eV and 531.4 eV confirm the existence of Bi-O-Bi bond of Bi_2_O_3_ in the partially oxidized Bi-ene-QDs [52]. The 1Ns at 406.06 eV, 407.16 eV, 399.38 eV, and 403.08 eV approve the presence of nitrogen which is corresponding to the core standard 1Ns band of nitryl [55]. Finally, the XPS data refer to the successful preparation of Bi-ene-QDs with the presence of surface oxygen element to create a thin laminate of Bi_2_O_3_ on the Bi-ene-QDs surface that protect it from being further oxidized and therefore, increase the stability of formed Bi-ene-QDs [54].

The SEM and TEM images of Bi-ene-QDs are clarified in Fig. 1d and e, correspondingly to provide good evidences for the synthesized material with semi-spherical structure and average particle size 6 nm. Further, SEM and TEM images indicated the randomness in the formation of Bi-ene-QDs with multiple sizes less than 10 nm [22]. The EDX analysis is given in Fig. 1f and denotes to the elemental percentage of fabricated Bi-ene-QDs as it contains bismuth, oxygen, nitrogen and carbon with percentages 78.87%, 16.48%, 1.10% and 3.55%, correspondingly [54].

The thermal stability of Bi-ene-QDs was examined by TGA thermogram as stated at Fig. 1g which indicates that the thermal decomposition were taken place by three stages. Primarily, at the first stage in the temperature extent 30–130 °C confirms a progressive mass decomposition with loss of 6.4% and weight 0.43 mg which is corresponding to the water evaporation [56]. The second stage is centered at the temperature extent 130–290 °C with weight loss 0.08 mg and percentage 1.2%, while the third stage is at temperature extent 290–578 °C with weight loss 0.62 mg and percentage 91.7%. These two stages were exhibited by the decomposition of bismuth metal oxidation in addition to the decomposition of exterior laminate of bismuth oxide at 290  C [57].

The BET analysis of Bi-ene-QDs was performed to to identify the specific surface area as illustrated by the N_2_ adsorption isotherm (Fig. 1h). Bi-ene-QDs showed H1 kind hysteresis loop which are corresponding to IV kind isotherm and indicating a mesoporous structure of Bi-ene-QDs with symmetric pore size. Bi-ene-QDs exhibited good surface area (157.78 m^2^/g). The pore size, beside pore volume values in Bi-ene-QDs were measured by BJH technique and found 7.23 nm and 0.378 cm^3^/g, correspondingly [58].

### Adsorptive capture studies of As(V) by Bi-ene-QD

The adsorptive capture of As(V) by Bi-ene-QDs was implemented by using batch removal operation that is depending on detecting the percentage of uptake as mentioned in the mathematical formula (1).

1 $$\text{\% }\mathbf{U}\mathbf{p}\mathbf{t}\mathbf{a}\mathbf{k}\mathbf{e} =\frac{{\mathbf{C}}_{\mathbf{o}}-{\mathbf{C}}_{\mathbf{e}}}{{\mathbf{C}}_{\mathbf{o}}} \times 100$$

And the *q*_*e*_ value for adsorptive capture of As(V) at equilibrium is detected from mathematical formula (2). As C_o_ and C_e_ are indicating primer and eventual concentrations of As(V), while V in L, and W in gm of Bi-ene-QDs [59].

2 $${{\varvec{q}}}_{{\varvec{e}}}=\frac{{{\varvec{C}}}_{{\varvec{o}}}-{{\varvec{C}}}_{{\varvec{e}}}}{{\varvec{W}}/{\varvec{V}}}$$

#### The ph influence on capture of As(V) by Bi-ene-QD and PZC

**Fig. 2:**
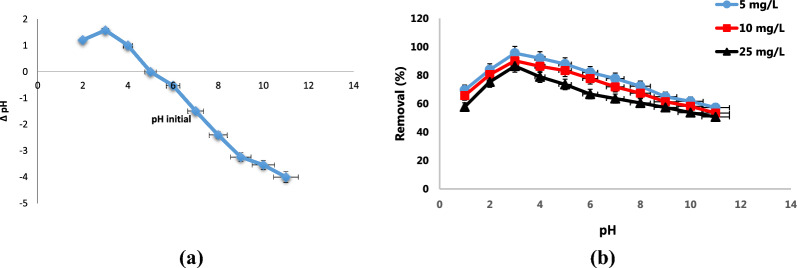
**a** Point of zero charge of Bi-ene-QDs. **b** Influence of pH on the abstraction of arsenate by (Bi-ene-QDs)

Point of zero charge (PZC) is a practical method for finding out the Bi-ene-QDs charge at multiple pHs extent as well as constant ionic strength as mentioned as illustrated in Fig. 2a. The PZC of Bi-ene-QDs = 5 and indicating that the Bi-ene-QDs charge below this point is dominantly positive, while posterior this point the Bi-ene-QDs charge is dominantly negative [60].

The influence of pH on effectiveness of adsorptive capture of As(V) by Bi-ene-QDs was examined in pH range 2.0–11.0 as clarified at Fig. 2b. It was observed that the removal percentage at pH 1.0 was very low yielding 69.61, 65.73, 57.75% for 5, 10 and 25 mg L^−1^ arsenate, correspondingly and these values were attained to maximum removal percentage at pH 3.0 exhibiting 95.54, 90.2 and 86.46 for 5, 10 and 25 mg L^−1^ arsenate, correspondingly. Moreover, with pH elevation, the percentage of adsorptive capture of As(V) by Bi-ene-QDs were decline to be 57.22, 53.41 and 50.68% by 5, 10 plus 25 mg L^−1^ As(V), correspondingly at pH 11.0. The existence of As(V) ions at pH < 2.3 is mainly in the formula of H_3_AsO_4_, while at pH extent 2.3–6.7 is mainly existing in the H_2_AsO_4_^−1^ formula and at pH > 6.7 it is present as HAsO_4_^−2^. According to the characterized PZC of Bi-ene-QDs, the surface is generally positive below pH 5.0. Therefore, Bi-ene-QDs could interact and complex with the negatively charged arsenate formulas to reach maximum adsorptive capture percentages of As(V) at pH 3.0. However, the decline in adsorptive capture percentages which might be attributed to the repulsion between arsenate ions the negative surface of Bi-ene-QDs at pH > 5.0 [61].

#### The Bi-eneQD mass influence on capture studies of As(V) by bi-ene-qds

**Fig. 3:**
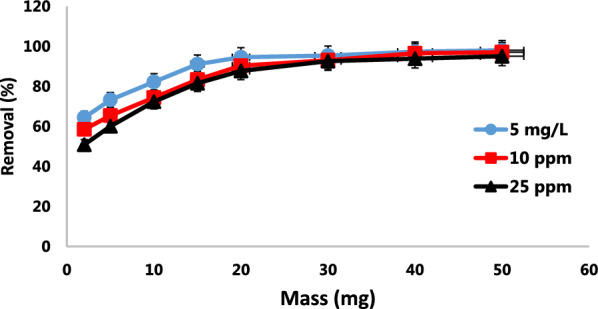
Effect of mass on As(V) sequestration by Bi-ene-QDs

The impact of Bi-ene-QDs weight on adsorptive capture percentages was examined by using 2.0–50.0 mg as demonstrated in Fig. 3. It was found that upon using 2.0 mg of Bi-ene-QDs, the adsorptive capture percentages of As(V) were 64.44, 58.51 and 50.89%, for  5, 10 plus 25 mg L^−1^ arsenate, correspondingly. While at 50.0 mg mass, the removal percentages were enhanced to be 98.04, 97.08 and 95.09% for 5, 10 and 25 mg L^−1^ As(V), correspondingly. The elevation in As(V) removal percentage by Bi-ene-QDs with enhancing Bi-ene-QDs weight is mainly due to the rising in number of attainable sites for adsorptive capture of As(V) upon increasing the Bi-ene-QDs weight [62].

#### The ionic strength and competing ions influence on capture of As(V) by Bi-ene-QD

**Fig. 4:**
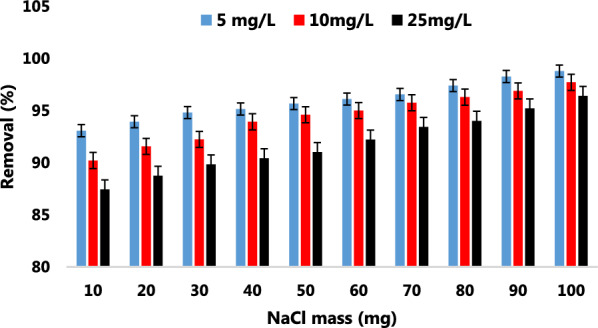
Effect of ionic strength on As(V) sequestration by Bi-ene-QDs

The ionic strength impact was examined by sodium chloride using various weights between 10–100 mg to investigate its influence on adsorptive capture of As(V) by Bi-ene-QDs as clarified in Fig. 4 [63]. The capture percentages were identified as 93.07 for 5 mg L^−1^ arsenate, 90.21 for 10 mg L^−1^ arsenate and 87.43% by 25 mg L^−1^ arsenate solution in the presence of 10 mg mass of sodium chloride. In addition, the adsorptive capture percentages of As(V) were found to progressively increase till reaching to the maximum values at 100 mg of sodium chloride providing 98.79 for 5 mg L^−1^ arsenate, 97.71 for 10 mg L^−1^ arsenate and 96.41% for 25 mg L^−1^ arsenate solution. This behavior confirms the selectivity of Bi-ene-QDs toward As(V), besides the occurrence of internal sphere complexation which can be detected by no effect of ionic strength on the removal percentage of As(V) [64, 65]. Moreover, the presence of sodium ion was found to overcome the competitive impact of the chloride ion and thus led to direct enhancement in the removal efficiency of As(V) by Bi-ene-QDs with increasing the ionic strength [66].

Moreover, the selective removal of As(V) by Bi-ene-QDs in presence of other competing ions as Mg(II), Ca(II) and Zn(II) was also investigated by using 1:1 mg L^−1^ ratio. The percentage removal of As(V) were corresponded to 86.83%, 84.44% and 81.15% when separately combined with Mg(II), Ca(II) and Zn(II), respectively. These values are very close to that characterized for removal of As(V) ions by Bi-ene-QDs (87.13%) in absence of these competing ions**.**

#### The interaction duration influence on capture of As(V) by Bi-ene-QD and kinetics study

**Fig. 5:**
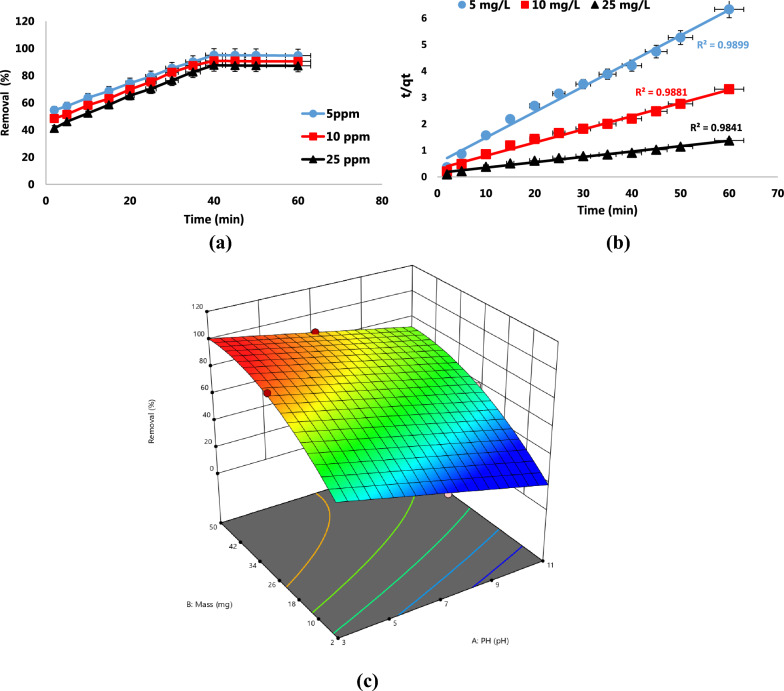
**a** Effect interaction duration for As(V) sequestration by Bi-ene-QDs. **b** *Pseudo second* order model for As(V) sequestration by Bi-ene-QDs. **c** RSM representation for removal of As(V) by Bi-ene-QDs

The interaction duration time at 2–60 min was utilized to characterize the optimum duration time for adsorptive capture of As(V) by Bi-ene-QDs as clarified at Fig. 5a. It was observed that at the first 2 min, As(V) was removed providing 54.58 for 5 mg L^−1^ arsenate, 48.49 for mg L^−1^ arsenate and 41.18% by 25 mg L^−1^ arsenate solution concentrations. Then the removal efficiency values were identified to reach the equilibrium at interaction duration 40 min with removal percentages corresponding to 95.05 for 5 mg L^−1^ arsenate, 90.86 for 10 mg L^−1^ arsenate, and 87.62% for 25 mg L^−1^ arsenate solution. The rapid adsorptive capture of As(V) by Bi-ene-QDs can be interpreted by the presence of attainable active sites and when these were loaded with As(V) ions, the removal capacity were slowed down till equilibration at 40 min [67].

Sorption kinetics is utilized to figure out the rate and removal mechanism(s) of arsenate ions via Bi-ene-QDs [68]. Therefore, five different kinetic patterns were examined including the pseudo-first order, pseudo-second-order, Intraparticle diffusion, power function and Elovich. The pseudo-first order pattern is relying on investigating the removal reactions in liquid–solid medium, as its supposes that permeation can take place from the interior of the nanosorbents and the variation at the adsorbate removal capacity with time is commensurate to the nanosorbent empty attainable and available sites [69] and the mathematical formula are mentioned in Table 2. As listed in Table 3 and Fig. 1Sa (Supplementary material), the R^2^ magnitudes were computed as 0.7328 for 5 mg L^−1^ arsenate, 0.8957 for 10 mg L^−1^ arsenate and 0.9171 for 25 mg L^−1^ arsenate solution. These are very low, besides the calculated removal magnitudes of As(V) (q_ecal_) were not matching with the experimental measured ones (q_eexp_) to indicating that this model is inconvenient to describe the adsorptive capture interaction of As(V) by Bi-ene-QDs [70].

**Table 2:** Several examined kinetic patterns for adsorptive sequestration of As(V) by Bi-ene-QDs

Kinetic model	Linear equation	Definition	Plot
*Pseudo*-first order	**ln (q**_**e**_** – q**_**t**_**) = ln q**_**e**_** – k**_**1**_**t**	q_e_ and q_t_ are the abstracted Arsenate magnitude (mg g^−1^) at stabilization and time t (min) and k_1_ is pseudo-first order rate constant (min^−1^)	**ln(q**_**e**_** − q**_**t**_**) versus time (t)**
*Pseudo*-second order	**t ⁄ q**_**t**_** = 1 ⁄ k**_**2**_**q**_**e**_^**2**^** + t ⁄ q**_**e**_	q_e_ and q_t_ are the abstracted arsenate magnitude (mg g^−1^) at stabilization and at time t (min) and k_2_ is the pseudo second order rate constant (g/(mg min)	**(t/q**_**t**_**) versus time (t)**
Intraparticle diffusion	**q**_**t**_** = k**_**id**_** t **^**½**^** + C**	k_id_ is the intraparticle diffusion rate constant (mg g^−1^ min^−1/2^) while C is the border laminate thickness (mg g^−1^)	**(q**_**t**_**) against (t**^**1/2**^**)**
Elovich	$q_{t} \, = \,\frac{1}{\beta }\,\ln \,(\alpha \beta )\; + \;\frac{1}{\beta }\ln \;t$	β is the stimulation energy,α is the primer abstraction average (mg g^−1^ min) and the exterior coverage of chemisorption	**(q**_**t**_**) versus ln t**
Power function	**lnQ**_**t**_** = lnb + K**_**f**_** (ln t)**	b power function rate constant, and k_f_ the rate coefficient (mg g^−1^ min^−1^)	**ln Qt verses ln t**

**Table 3:** Computed kinetic magnitudes for adsorptive sequestration of As(V) by Bi-ene-QDs

Kinetic model	As (mg L^−1^)		
	5	10	25
***Pseudo*-first order** q_e_ (mg g^−1^)(exp.) q_e_ (mg g^−1^)(calc.) k_1_ (min.^−1^) R^2^	6.35 9.51 0.062 0.7328	12.61 18.17 0.069 0.8957	31.46 43.81 0.061 0.9171
***Pseudo-*second order** q_e_ (mg g^−1^)(exp.) q_e_ (mg g^−1^)(calc.) k_2_ (g mg^−1^ min.^−1^) R^2^	10.35 9.50 0.0179 0.9899	20.08 18.17 8.21 *10^–3^ 0.9881	49.26 43.81 2.76 × 10^–3^ 0.9841
**Intraparticle diffusion** K_id_(mg.g^−1^ min^−1/2^) C R^2^	0.762 4.17 0.9607	1.60 7.05 0.9539	4.28 13.89 0. 9626
**Elovich** α (mg g^−1^ min^−1^) β (mg g^−1^) R^2^	18.42 0.699 0.9054	21.47 0. 331 0.9066	30.99 0.124 0.9166
**Power function** b K_f_ (mg g^−1^ min^−1^) R^2^	4.35 0.194 0.9356	7.54 0.221 0.9381	15.71 0.258 0.9551

The pseudo-second-order is relying on a chemical removal process [71] and the mathematical formula is stated at Table 2. As listed in Fig. 5b, the R^2^ were found high as 0.9899 for 5 mg L^−1^ arsenate, 0.9881 for 10 mg L^−1^ arsenate and 0.9841 for 25 mg L^−1^ arsenate solutions. Moreover, the (q_ecal_) measurements 9.50, 18.17 and 43.81 (mg g^−1^) are relatively close to the detected experimental values (q_eexp_) as 10.35 (mg g^−1^) for 5 mg L^−1^ arsenate, 20.08 (mg g^−1^) for 10 mg L^−1^ arsenate and 49.26 (mg g^−1^) for 25 mg L^−1^ arsenate, correspondingly to confirming that this pattern is suitable to depict the adsorptive capture of As(V) by Bi-ene-QDs. In addition, k_2_ were found to descended from 0.0179 (g/mg min) by 5 mg L ^−1^ arsenate to 0.00276 (g/mg min) by 25 mg L ^−1^ to confirm that Bi-ene-QDs remove low As(V) concentrations faster [72]. The expression of intraparticle diffusion is relying on two main concepts, the primer one is the diffusion of As(V) ions into Bi-ene-QDs, while the second concept supposes the As(V) pervasion into the Bi-ene-QDs pores [70] and the mathematical formula is provided in Table 2. The computed R^2^ by this pattern were 0.9607 for 5 mg L^−1^ arsenate, 0.9539 for 10 mg L^−1^ arsenate, 0.9626 for 25 mg L^−1^ arsenate concentrations. Besides, it was confirmed that with elevating the concentration of As(V), there was an enhancement in the diffusion rate constant (k_id_) and to refer to faster intraparticle As(V) pervasion operation at elevated concentrations [67]. According to Fig. 1Sb (Supplementary material), the intraparticle diffusion could not be taken as the main rate predominant stage as the lines of linear plot are not going through the origin [73].

Furthermore, the power function pattern was also examined to provide the relation among As(V) removal by Bi-ene-QDs and time [74] with the mathematical formula is given in Table 2. As stated at Table 3 and Fig. 1Sc (Supplementary material), it was observed that with increasing As(V) concentration, the power function rate constant (b) magnitude was elevated which mean at higher concentration the removal rate of As(V) was faster intraparticle As(V) pervasion operation at elevated concentrations [67]. The Elovich model is mainly applied for depicting the asymmetric surface as the most compatible pattern to interpret chemisorption interactions [73] with the mathematical formula is stated in Table 2. As outlined in Table 3 and Fig. 1Sd (Supplementary material), the computed R^2^ were 0.9054 for 5 mg L^−1^ arsenate, 0.9066 for 10 mg L^−1^ arsenate and 0.9166 for 25 mg L^−1^ arsenate solution. Besides, the (α) magnitude was detected to increase by enhancing the As(V) concentration as this chemisorption action interpret the occurrence of internal sphere complexation between As(V) ions and Bi-ene-QDs at pH 3.0. While, (β) magnitudes were found to decrease upon enhancing the As(V) ions concentration due to the decline in the attainable sites on Bi-ene-QDs.

Eventually, according to R^2^ magnitudes, the pseudo-second order with chemisorption interaction behavior is the most convenient pattern to depict the adsorptive capture of As(V) onto Bi-ene-QDs.

To study the combined effective factors such as pH, mass dosage, and time in the removal process, a quadratic model Box Behnken was applied. The ANOVA results were evaluated, and the P-value was less than 0.05 indicating the model terms are significant. The model F-value 179.03 implies the model is significant. The predicted R^2^ 0.9451 is in a reasonable agreement with the adjusted R^2^ 0.9889; to confirm that the difference is less than 0.2. the final equation in terms of coded factors is given by the following expression: % Removal = 87.68–10.12A + 18.38B + 8.00C + 2.50AB + 6.25AC-6.25BC-13.29B^2^-4.04C^2^ where A is the pH, B is the mass dosage and C is the time. The response surface plots for the removal process are illustrated in Fig. 5c to refer that the pH, mass dosage and time interactions have a high effect on the removal process.

#### The As(V) concentration influence on capture by Bi-ene-QD and adsorption isotherms

**Fig. 6:**
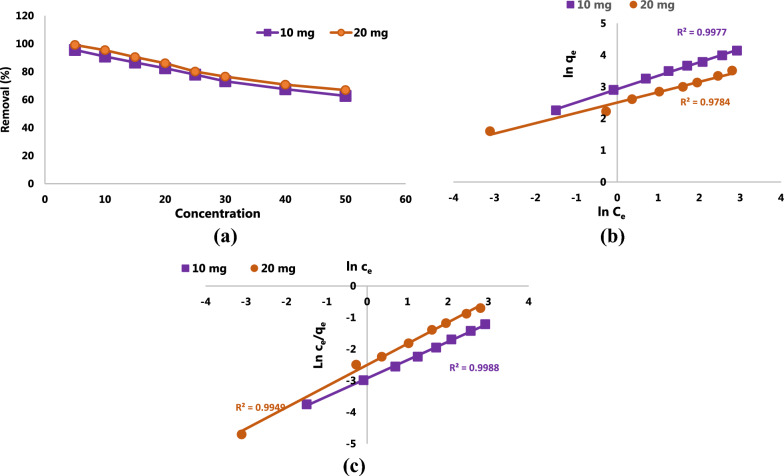
**a** The arsenate concentration influence on As(V) sequestration by Bi-ene-QDs. **b** Freundlich model for As(V) sequestration by Bi-ene-QDs. **c.** Redlich-Peterson model for As(V) sequestration by Bi-ene-QDs

The influence of various concentrations of As(V) on its removal by Bi-ene-QDs was examined using concentrations in the range 5–50 mg L^−1^ in presence of 10 and 20 mg masses of Bi-ene-QDs. As illustrated in Fig. 6a. the removal percentages were 95.52 and 99.10% at 5 mg L^−1^ by 10 mg and 20 mg of Bi-ene-QDs, correspondingly. While, with increasing arsenate concentration, the removal percentage declined to 62.71% for 10 mg and 66.86% for 20 mg Bi-ene-QDs via 50 mg L^−1^ arsenate. On the other hand, the (q_e_) was enhanced with elevating As(V) concentration. Subsequently, the depression in removal percentage with increasing As(V) concentration is due to the decline in the number of attainable sites on Bi-ene-QDs [60]. While the increasing of arsenate removal magnitudes (q_e_) with increasing arsenate concentration is due to the elevation of As(V) mobile power that facilitate faster As(V) spreading through Bi-ene-QDs attainable positions [62].

In order to investigate the interaction between arsenate ions and Bi-ene-QDs, adsorption isotherms were studied. The studied adsorption isotherms are Langmuir, Freundlich, Dubinin–Rasushkevich (D-R), Redlich-Peterson in addition to Temkin. Firstly, the Langmuir isotherm hypothesis suggested the removal process to take place on attainable sites at adsorbent with symmetric energy distribution without combination among adsorbed species and the mathematical formula is stated at Table 4 [75]. As provided in Fig. 2Sa (Supplementary material) and Table 5, the R^2^ magnitudes were identified as 0.9697 and 0.9552, besides the (q_max_) were found to correspond to 68.97 and 70.42 (mg/g) for 10 and 20 mg of Bi-ene-QDs. These are higher than the other reported applied nanocomposites for removal of As(V) from water as mentioned in Table 7 to assure the excellent removal efficiency of Bi-ene-QDs. The Langmuir separation constant (R_L_) magnitudes are detected to be (0.3887–0.0598) and (0.3225–0.0454) by 10 and 20 mg of Bi-ene-QDs to refer to the appropriate removal operation as R_L_ magnitudes is greater than zero and less than one to confirm a spontaneous process [62].

**Table 4:** Several examined isotherm models for adsorptive sequestration of As(V) by Bi-ene-QD

Adsorption isotherm models	Equation Linear form	Parameter definition	Plot
Langmuir	Ce/qe = (1/qmax) k_1_ + Ce/qmax b RL = 1/1 + b C°	C_o_ and C_e_ the initial and equilibrium concentrations (mg L^−1^), respectively. q_e_ is the magnitude of abstracted Arsenate at equilibrium (mg g^−1^). q_max_ (mg g^−1^) the maximum abstracted arsenate which is used to determine the abstraction energy and the abstraction efficiency besides b (L mg^−1^) is Langmuir constants	C_e_/q_e_ versus C_e_
Freundlich	lnqe = ln k_f_ + 1/n ln Ce	q_e_ is the magnitude of up taken Arsenate which is corresponding to the equilibrium concentration of Arsenate in solution and C_e_ is the equilibrium Arsenate concentration. K_F_ (mg g^−1^) is Freundlich constant, n is the intensity of the adsorbent	ln q_e_ versus ln C_e_
Temkin	q_e_ = (RT/b_T_) ln a_T_ + (RT/b_T_) ln c_e_ q_e_ = B ln a_T_ + B ln C_e_ B = $\frac{\mathbf{R}\mathbf{T}}{\mathbf{b}\mathbf{t}}$	b_T_ (mg L^−1^) is the Temkin isotherm constant, a_T_ (L g^−1^) is the Temkin isotherm equilibrium binding constant and B is constant represents the heat of arsenate abstraction reaction (J/mol)	q_e_ versus ln C_e_
Redlich-Peterson isotherm	${\varvec{l}}{\varvec{n}}\left( {{\varvec{K}}}_{{\varvec{R}}{\varvec{P}} } \frac{{{\varvec{C}}}_{{\varvec{e}}}}{{{\varvec{q}}}_{{\varvec{e}}}}-1\right)={\varvec{g}}{\varvec{l}}{\varvec{n}}{{\varvec{c}}}_{{\varvec{e}}}+{\varvec{l}}{\varvec{n}}{{\varvec{a}}}_{{\varvec{R}}{\varvec{P}}}$	C_e_ is the initial and equilibrium concentrations (mg.L^−1^), respectively. q_e_ is the magnitude of abstracted arsenate at equilibrium (mg g^−1^) as a_RP_ (Lg.mg^−g^) and K_RP_(L.g^−1^) are constants	ln c_e_/q_e_ versus ln c_e_
Dubinin–Radushkevich (D–R)	ln q_e_ = ln q_s_ – (K_ad_ ε^2^) ε = RT ln (1 + 1/Ce) Es = $\frac{1}{\sqrt{2 \mathbf{K}\mathbf{a}\mathbf{d}}}$	K_ad_ the D–R isotherm constant which is related to the abstraction free energy per mole of the arsenate (mol^2^/kJ^2^). q_s_ (mg g^−1^) is the theoretical fullness capacity ε is the Polanyi potential which is based on equilibrium, R is the universal gas constant (8.314J/mol K^−1^) and T absolute temp. Kelvin	ln q_e_ versus ε^2^

**Table 5:** Computed isotherm magnitudes for adsorptive sequestration of As(V) by Bi-ene-QD

Isotherm model	Parameters	Mass of Bi-ene-QD	
		10 mg	20 mg
Langmuir	q_max_ (mg/g) b (L mg^−1^) R_L_ R^2^	68.96 0.314 (0.389—0.0598) 0.9697	70.42 0.420 (0.323–0.0454) 0.9552
Freundlich	n K_f_ (L. mg^−1^) R^2^	2.37 18.65 0.9977	3.07 24.44 0.9784
Temkin	a_T_ (L. g^−1^) b_T_ ( J/mol) B R^2^	6.14 209.32 11.83 0.9439	24.45 268.41 9.23 0.8507
Redlich-Peterson isotherm	g a_RP_ R^2^	0.5783 0.0536 0.9988	0.6741 0.0409 0.9949
Dubinin-Radushkevich	q_s_ (mg/g) K_ad_ (mol^2^/ j^2^) E_s_ (kJ mol^−1^) R^2^	39.74 9* 10^–8^ 2.3 0.7358	19.74 2* 10^–8^ 5 0.6389

The Freundlich isotherm depicts asymmetric surface, besides the exponential apportionment of attainable positions and their energies. Further, this pattern hypothesis the multiple laminates coverage at asymmetric adsorption attainable positions at adsorbent as stated at Table 4 [75]. According to Fig. 6b and Table 5, the R^2^ magnitudes were found 0.9977 and 0.9784, while the n magnitudes are 2.37 and 3.07 for 10 and 20 mg of Bi-ene-QDs, which interpret the favorable removal operation of As(V) by Bi-ene-QDs to indicate an appropriate removal process because the n-values are between 2–10 [76].

Temkin isotherm hypothesis is based on the coverage increase onto adsorbent surface, while the adsorption heats of molecules decrease linearly to result in a uniform distribution of binding energies up to the maximum. This favors an electrostatic attraction removal mechanism depending on chemisorption process [77], while the mathematical formula is listed at Table 4. According to Fig. 2Sb (Supplementary material) the R^2^ magnitudes were 0.9439 and 0.8504 for 10 and 20 mg of Bi-ene-QDs, besides the other parameters are stated at Table 5.

The Redlich-Peterson isotherm is multilateral isotherm which is based on an empirical formula of a combination between Freundlich along with Langmuir patterns for depicting the symmetric and asymmetric systems as given by the mathematical formula in Table 4. The g-exponent is at the extent (0 ≤ g ≤ 1), and as the g is equal to one the pattern is corresponding to Langmuir pattern, and when g is equal to zero the pattern is turned to linear pattern [70]. According to Fig. 6c and Table 5, the R^2^ magnitudes are 0.9988 and 0.9949, while g magnitudes are 0.5783 and 0.6741 for 10 and 20 mg of Bi-ene-QDs.

The D-R isotherm hypothesis was also investigated in this work according to the mathematical formula listed in Table 4 [63]. According to Fig. 2Sc (Supplementary material) and Table 5, the R^2^ magnitudes were 0.7358 and 0.6389 for 10 and 20 mg of Bi-ene-QDs which are very low value indicating poor convenience of this isotherm, besides the E_s_ measurements were found 2.3 and 5 (kJ /mol) for 10 and 20 mg of Bi-ene-QDs to refer to physisorption removal of As(V) onto Bi-ene-QDs because the Es values are less than 8 (kJ/mol) [62].

Eventually, according to the previously mentioned data and R^2^ magnitudes, the most appropriate models to depict the interaction of As(V) ions and Bi-ene-QDs are Freundlich in addition to Redlich-Peterson isotherms.

#### The temperature influence on capture of As(V) Bi-ene-Qd

**Fig. 7:**
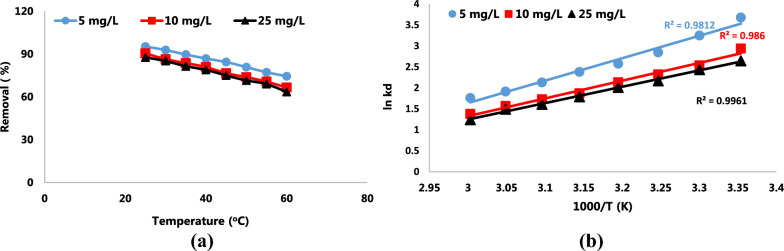
**a** Effect of temperature on As(V) sequestration by Bi-ene-QDs. **b** Van’t Hoff graph for As(V) sequestration by Bi-ene-QDs

For investigating the temperature influence on the adsorptive capture of As(V) by Bi-ene-QDs, the temperature range 25–60 °C was selected and applied. As clarified in Fig. 7a, the removal percentages were declined with elevating the temperature. At 25 °C the adsorptive capture percentages of As(V) were 95.2 for 5 mg L^−1^ arsenate, 90.44 for 10 mg L^−1^ arsenate and 87.59% for 25 mg L^−1^ arsenate. While at 60  C, these were found 74.4 for 5 mg L^−1^ arsenate, 66.53 for 10 mg L^−1^ arsenate and 63.399% for 25 mg L^−1^ arsenate. The thermodynamic characteristics of adsorptive capture of As(V) by Bi-ene-QDs, thermodynamic parameters as (ΔH°), (ΔS°), besides (ΔG°) were estimated from Van’t Hoff graph from Eqs. (3, 4, 5 and 6) [73].

3 $$\text{ln Kd }= \Delta \text{S}^\circ /\text{R}-\Delta \text{H}^\circ /\text{RT}$$

4 $$\Delta \text{G}^\circ =-\text{RT lnKD}$$

5 $$\text{KD}=\text{qe}/\text{ce}$$

where K_D_ is the sorption equilibrium constant (L/g), C_e_ arsenate concentration at equilibrium (mg/L) the, q_e_ the sequestered arsenate by Bi-ene-QDs (mg/g). According to Van’t Hoff graph, when lnK_D_ verses 1/T as provided in Fig. 7b, (ΔS°) as well as (ΔH°) values were computed and compiled in Table 6. Thus, the negative estimates of (ΔH°) and (ΔG°) refers to exothermic and spontaneous interaction between As(V) ions and Bi-ene-QDs, while negative ΔS° values elucidates a decrease in entropy leading to less randomized system due to the action of As(V) adsorptive capture onto Bi-ene-QDs [78].

**Table 6:** Thermodynamic parameters for the arsenate abstraction by (Bi-ene-QDs) at several temperatures

As(V) mg/L	Adsorption Thermodynamic Parameters																	
	K_d_ (L/g)								−ΔG° (KJ/mol)								ΔS° (J/mol.K)	ΔH° (KJ/mol)
	298 K	303 K	308 K	313 K	318 K	323 K	328 K	333 K	298 K	303 K	308 K	313 K	318 K	323 K	328 K	333 K		
**5**	39.67	25.78	17.23	13.15	10.82	8.42	6.77	5.81	9.12	8.19	7.29	6.70	6.30	5.72	5.21	4.87	−119.75	−44.46
**10**	18.91	12.64	10.2	8.46	6.51	5.62	4.81	3.98	7.28	6.39	5.95	5.56	4.95	4.64	4.28	3.82	−94.49	−35.17
**25**	14.12	11.43	8.75	7.48	5.98	5.00	4.45	3.46	6.56	6.14	5.56	5.24	4.73	4.33	4.07	3.44	−86.99	−32.45

### Recycling process of Bi-ene-QD

Bi-ene-QDs were subjected to adsorptive capture of As(V) from water for several cycles to clarify its capacity, environmental and economic implementations. A 0.1 M hydrochloric acid solution was utilized for desorption of As(V) from Bi-ene-QDs. The removal percentages of As(V) were as following at the first cycle was 88.73%, while the second cycle was 74.75%, and the third cycle was 63.73% by using 5 mg L^−1^ arsenate. While at application of 25 mg L^−1^ concentration the removal percentages were confirmed by the first cycle as 76.35%, while the second cycle was 62.14%, and the third cycle was 51.47%. According to previously mentioned results, it can be concluded that Bi-ene-QDs exhibited a reasonable recyclability effectiveness and environmental stabilization. On the other hand, the decline in removal percentage after each cycle can be interpreted by embargo of the attainable sites on the Bi-ene-QDs due to incomplete desorption of arsenate by hydrochloric acid [79]. It is evident that HCl as a strong acid was found invalid in the regeneration process of Bi-ene-QDs based on the low percentage removal values of As(V), especially after the second and third cycles. Therefore, another recycling procedure was performed by using 100 mL 0.1 M NaCl as a neutral and recovery solution at each cycle. Three successive cycles were performed and the regenerated Bi-ene-QDs materials were employed to figure out their efficiency in removal of the same three As(V) concentrations, viz. 5, 10 and 25 mg L^−1^. The firstly recycled Bi-ene-QDs provided excellent removal values of As(V) by providing 91.97% (5 mg L^−1^), 87.72% (10 mg L^−1^) and 83.69% (25 mg L^−1^). The secondly treated Bi-ene-QDs exhibited good removal values of As(V) giving 87.07% (5 mg L^−1^), 84.02% (10 mg L^−1^) and 78.89% (25 mg L^−1^), while the third recycled material afforded 83.76% (5 mg L^−1^), 82.21% (10 mg L^−1^) and 77.15% (25 mg L^−1^). Therefore, it is concluded that the regeneration of Bi-ene-QDs material can be mainly favored by using neutral solutions as NaCl.

### Bi-ene-qds application in capture of as(V) from actual water systems

A study was performed to validate the potential application of Bi-ene-QDs for removal of arsenate from several water specimens as tap, sea and wastewater. Therefore, 10 mg of Bi-ene- QDs was used to remove As(V) from 20 mL solution (5, 10 and 25 mg L^−1^) by the batch mode. The arsenate removal from tap water were 95.21% for 5 mg L^−1^ arsenate, 92.22% for 10 mg L^−1^ arsenate and 91.94% for 25 mg L^−1^ arsenate. While the adsorptive capture of arsenate onto Bi-ene-QDs from sea water sample provided 94.61% for 5 mg L^−1^ arsenate, 91.13% for 10 mg L^−1^ arsenate and 90.48% for 25 mg L^−1^ arsenate. Further, arsenate in wastewater exhibited removal percentages 94.38, 91.22 and 91.48% removal percentages from the three applied arsenate concentrations. Eventually, one can conclude that the assembled and investigated Bi-ene-QDs nanosorbent was found highly successful and effective in removal of As(V) pollutant from aqueous medium as well as actual water systems with the achievement of more than 90.0% removal efficiency.

## Conclusion

Bi-ene-QDs nanosorbent was prepared from Bi(NO_3_)_3_·5H_2_O by green and facile one step hydrothermal reaction. The FT-IR analysis confirmed a vibrational peak at 3394 cm^−1^ for the hydroxyl group in the Bi-ene-QDs exterior surface, while the corresponding FTIR characterization of As(V)@Bi-ene-QDs referred to the appearance of As-O stretching vibration based on possible adsorptive capture of As(V) onto Bi-ene-QDs. Moreover, the XRD referred to the crystalline structure of The characterized Bi-ene-QDs material exhibited semi-spherical crystalline formula with average particle size 6.0 nm and surface area 157.78 m^2^/g. As(V) ions were optimally captured by 10 mg mass of Bi-ene-QDs at pH 3.0 and 40 min interaction duration. Internal sphere complexation was found to take place between As(V) and Bi-ene-QDs based on the elevated adsorptive capture percentage by enhancing the solution ionic strength. The adsorptive capture of As(VI) by Bi-ene-QDs followed an exothermic and spontaneous reactions, while the *pseudo*-second order pattern was identified as the most compatible kinetic model to depict this interaction process. Further, the Freundlich plus the Redlich-Peterson isotherms were found the most adequate patterns to depict the adsorptive capture reaction of As(VI) by Bi-ene-QDs. When, Bi-ene-QDs were applied to remove As(V) ions from actual water specimens, excellent results were accomplished according to adsorptive capture percentages as 94.61%, 95.21% and 94.38% from sea, tap and wastewater, correspondingly. Therefore, the current study presents Bi-ene-QDs as a novel and efficient nanosorbent for effective adsorptive capture of As(V) pollutant from aqueous medium as well as actual water systems compared versus previously reported materials as listed in Table 7 [58, 61, 73, 80–83].

**Table 7:** Comparisons of the adsorptive sequestration of As(V) by Bi-ene-QD versus other materials

Adsorbent	pH	q_max_ (mg/g)	References
MIL-125(Ti)	3.0	46.34	[58]
Porous flowered graphene oxide-lanthanum fluoride (GO-LaF)	6.0	18.52	[61]
A33E	6.0	34.41	[73]
A33E-Nd(III)	6.0	56.01	[73]
Cerium oxide modified activated carbon	5.0	43.6	[80]
Hydrous cerium oxide–graphene composite	7.0	41.31	[81]
gGO-Gd_2_O_3_	4	36.77	[82]
La-modified ceramic material	6.8	22.90	[83]
Bi-ene-QDs (10 mg)	3	68.96	This study
Bi-ene-QDs (20 mg)	3	70.42	This study

## Supplementary information

Supplementary Material 1

## Data Availability

No datasets were generated or analysed during the current study.
