# Supplementary material for: Effective capture of As(V) from water by a facile one step hydrothermal synthesized of 2-D bismuthene quantum dots nanosorbent
Source: BMC Chem. 2024 Oct 17;18(1):202. doi: 10.1186/s13065-024-01308-x (PMC11487798; doi:10.1186/s13065-024-01308-x)
Supplement: Supplementary file 1 — Supplementary Material 1 [file 13065_2024_1308_MOESM1_ESM.docx]

**Effective capture of As(V) from water by a facile one step hydrothermal synthesized of 2-D bismuthene quantum dots nanosorbent**

**Saad S. M. Hassan^a^, Mohamed E. Mahmoud^b*^, Rana M. Tharwat^a^, Amir M. Abdelfattah^b^**

**^a^** Faculty of Science, Chemistry Department, Ain Shams University, P.O. Box 80205, Cairo, Egypt

**^b^** Faculty of Sciences, Chemistry Department, Alexandria University, Moharem Bey, Alexandria, Egypt. E.mail: memahmoud10@yahoo.com

**Chemicals and solutions**

Bi(NO_3_)_3_·5H_2_O (FW 485.07 g.mol^-1^ & 98.5%), disodium hydrogen arsenate (Na_2_HAsO_4_) (FW 312.01 g.mol^-1^ & 98%), (NH_4_)_2_MoO_4_) (FW 200.06 gmol^-1^ & 99.98%), L-Ascorbic acid (FW 176.124 g.mol^-1^ & 30%) purchased from Merckmillipore, Germany. NaOH (FW 40 g.mol^-1^ & 98%), sodium chloride (F.W 58.44 gmol^-1^ & purity ˃100%), hydrochloric acid (FW 36.46 gmol^-1^ & 37%) were obtained from lobachemie PVT, LTD, India.

**Figure 1Sa.** *Pseudo first* order model for As(V) sequestration by Bi-ene-QDs

**Figure 1Sb.** Intraparticle diffusion model for As(V) sequestration by Bi-ene-QDs

**Figure 1Sc.** Power function for As(V) sequestration by Bi-ene-QDs

**Figure 1Sd.** Elovich model for As(V) sequestration by Bi-ene-QDs

**Figure 2Sa.** Langmuir model for As(V) sequestration by Bi-ene-QDs

**Figure 2Sb.** Temkin model for As(V) sequestration by Bi-ene-QDs

**Figure 2Sc.** D-R model for As(V) sequestration by Bi-ene-QDs
